# Redox-Related Epigenetic Mechanisms in Glioblastoma: Nuclear Factor (Erythroid-Derived 2)-Like 2, Cobalamin, and Dopamine Receptor Subtype 4

**DOI:** 10.3389/fonc.2017.00046

**Published:** 2017-03-30

**Authors:** Matthew Scott Schrier, Malav Suchin Trivedi, Richard Carlton Deth

**Affiliations:** ^1^Department of Pharmaceutical Sciences, College of Pharmacy, Nova Southeastern University, Fort Lauderdale, FL, USA

**Keywords:** cobalamin, D_4_ dopamine receptor, glioblastoma, Nrf2, DNA methylation, glutathione, methionine synthase, transsulfuration

## Abstract

Glioblastoma is an exceptionally difficult cancer to treat. Cancer is universally marked by epigenetic changes, which play key roles in sustaining a malignant phenotype, in addition to disease progression and patient survival. Studies have shown strong links between the cellular redox state and epigenetics. Nuclear factor (erythroid-derived 2)-like 2 (Nrf2) is a redox-sensitive transcription factor that upregulates endogenous antioxidant production, and is aberrantly expressed in many cancers, including glioblastoma. Methylation of DNA and histones provides a mode of epigenetic regulation, and cobalamin-dependent reactions link the redox state to methylation. Antagonists of dopamine receptor subtype 4 (D_4_ receptor) were recently shown to restrict glioblastoma stem cell growth by downregulating trophic signaling, resulting in inhibition of functional autophagy. In addition to stimulating glioblastoma stem cell growth, D_4_ receptors have the unique ability to catalyze cobalamin-dependent phospholipid methylation. Therefore, D_4_ receptors represent an important node in a molecular reflex pathway involving Nrf2 and cobalamin, operating in conjunction with redox status and methyl group donor availability. In this article, we describe the redox-related effects of Nrf2, cobalamin metabolism, and the D_4_ receptor on the regulation of the epigenetic state in glioblastoma.

## Introduction

Gliomas are the most common primary central nervous system (CNS) tumors in adults, accounting for 78% of all primary malignant CNS tumors (1). Glial tumors are classified based on the type of glial cell giving rise to the tumor as oligodendrogliomas (oligodendrocytes), ependymomas (ependymal cells), and astrocytomas (astrocytes). Astrocytomas, which account for most cases of glioma (1), can be ranked as low-grade (WHO grade I–II) or high-grade (WHO grade III–IV), depending on their clinical behavior and genetic features (2).

High-grade glioma (WHO grade IV) is typically divided into four subtypes: classical, neural, proneural, and mesenchymal (3). Classical glioblastoma presents with chromosomal abnormalities, including epidermal growth factor receptor (EGFR) mutation and/or increased EGFR expression, but not protein 53 (p53) mutations (3). The neural subtype shares some similarities with the classical subtype, but frequently has p53 mutations and is positive for certain neuronal cell markers, such as neurofilament light peptide (NEFL) and gamma-aminobutyric acid receptor subunit α-1 (3). Proneural glioblastoma is associated with elevated plasma membrane levels of platelet-derived growth factor receptor and often have isocitrate dehydrogenase 1/2 (IDH1/2) mutations. The mesenchymal subtype uniquely displays activation of inflammatory mediators, especially NF-κB (3).

Surgical removal of primary glioblastoma is the preferred treatment option to prolong survival. While life expectancy can be prolonged by several months, the outcome is poor in the majority of patients who undergo resection because the tumor often continues to progress (4). At this time, there are two main non-surgical treatment protocols for glioblastoma. The first protocol comprises temozolomide, an orally administered DNA alkylating drug given in combination with radiation as first-line therapy (5). The second protocol comprises administration of the anti-angiogenic drug bevacizumab in patients with recurrent glioblastoma (6).

Despite treatment, the median survival time of patients with glioblastoma is between 9 and 12 months (1). A major cause of treatment failure is persistence of the epigenetic signatures, which define the malignant state (7). Epigenetic regulation provides dynamic control over gene expression. The methylation states of DNA and histones, which are influenced by intracellular S-adenosylmethionine (SAM) levels, are crucial determinants of this mode of regulation. DNA methylation at CpG sites promotes heterochromatin formation and gene silencing. Additionally, methylation of specific residues on the tails of histones, such as histone 3 lysine residue 9 (H3K9me) and histone 3 lysine residue 27 (H3K27me), can promote transcriptional silencing. However, other histone tail methylation marks, such as H3K4me, can lead to less dense nucleosome packing, thereby increasing promoter accessibility to transcription factors and favoring gene transcription.

Cancer is marked by genome-wide hypomethylation, chromosomal aberrations, and active transposable elements. Furthermore, cancer often presents with promoter hypomethylation or hypermethylation of genes expressing the components of major signaling pathways. The epigenetic landscape of glioblastoma includes differential expression patterns of long non-coding RNAs, such as increased HOTAIR (8). LncRNAs cooperate with proteins to modulate gene expression. Another typical epigenetic feature of glioblastomas is upregulation of EZH2 (9), the catalytic component of polycomb repressive complex 2, that methylates H3K27 (histone 3 lysine residue 27) (9), leading to condensation of nucleosomes. Paradoxically, several proteins that promote gene expression are also overexpressed, such as BRD2 (bromodomain-containing 2) and BRD4 (10). BRD2 and BRD4 are members of the bromodomain and extra-terminal (BET) family of proteins, which recognize acetylated lysine residues on histones and promote transcription of the histone-associated genes. Additionally, certain lysine-specific demethylases (KDMs) and 5-methylcytosine hydroxylation enzymes, called ten-eleven translocation (TET) enzymes, are inhibited by oncometabolites, such as 2-hydroxyglutarate (2-HG) and succinate (11).

Nuclear factor (erythroid-derived 2)-like 2 (Nrf2) is a transcription factor activated during oxidative stress that upregulates the expression of genes involved in antioxidant production. Glioblastoma and numerous other cancers are characterized by aberrant Nrf2 expression. Methylcobalamin (MeCbl) and 5′-deoxyadenosylcobalamin (AdoCbl)—the active forms of cobalamin (vitamin B12)—play central roles in regulating DNA and histone methylation by serving as cofactors for methionine synthase (MS) and methylmalonyl-CoA mutase (MCM), respectively. Their levels are sensitive to the cellular antioxidant status, making them responsive to Nrf2. The dopamine receptor subtype 4 (D_4_ receptor) is a G protein-coupled receptor (GPCR) with neurotransmission and metabolic roles, which include effects on the redox and methylation states. D_4_ receptors are expressed in glioblastoma and D_4_ receptor antagonists inhibit the growth of glioblastoma stem cells (12), although the precise relationship between Nrf2 and D_4_ receptors has not yet been established. This article is the first to explore the relationships between Nrf2, cobalamin, and D_4_ receptors in cancer, specifically in glioblastoma. Recognition of these relationships opens new opportunities for identifying novel drug targets.

## The Role of Nrf2 in Cancer and Glioblastoma

**Figure 1:**
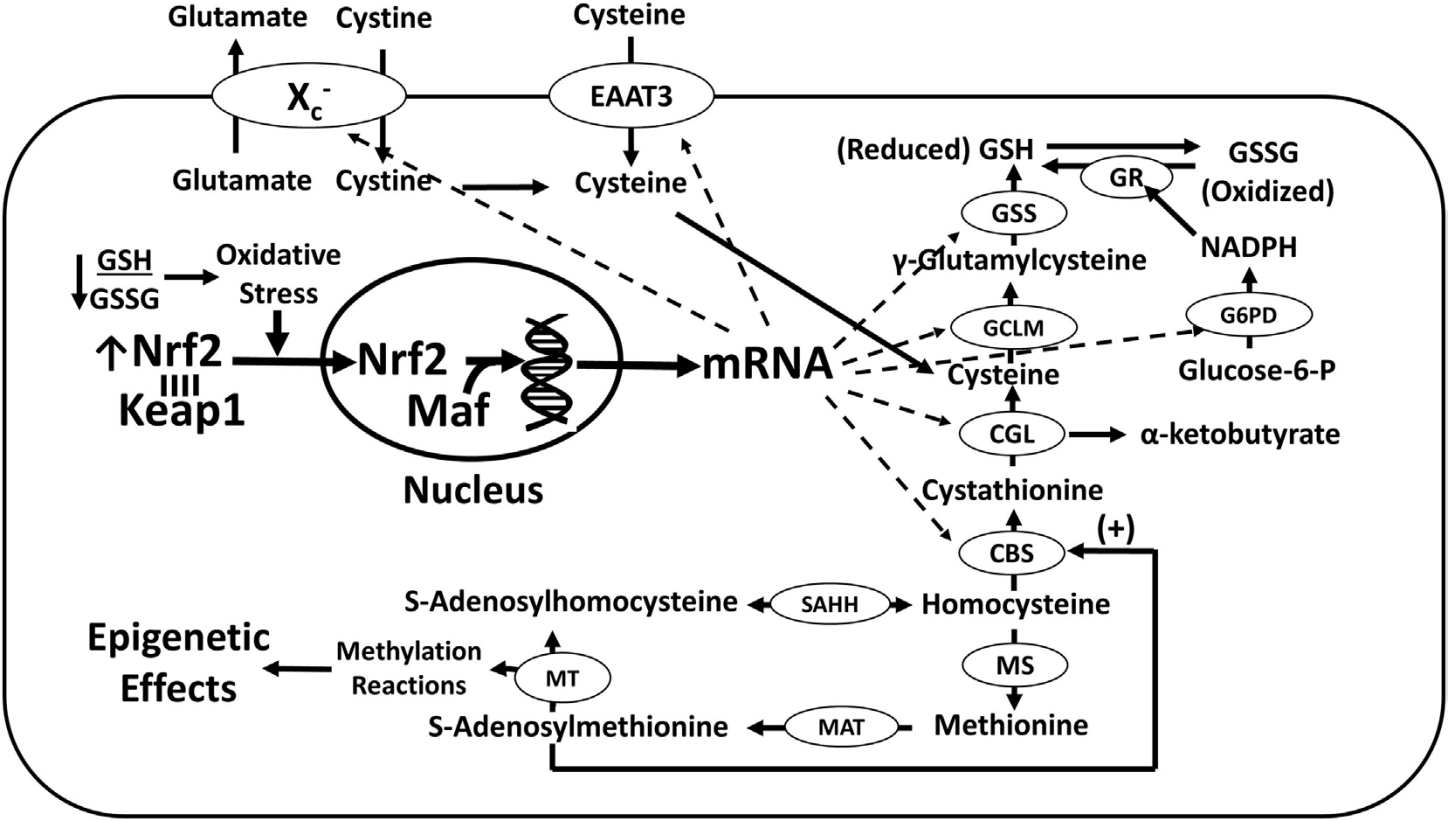
**Nrf2-dependent transcription of genes promoting GSH synthesis**. Oxidative stress induces the dissociation of Nrf2 from cytosolic Keap1, allowing its relocation to the nucleus where it augments the transcription of multiple genes directly or indirectly involved in GSH synthesis (dashed arrows), thereby increasing antioxidant capacity. Nrf2 activation of GSH synthesis negatively affects the methylation capacity by promoting transsulfuration of homocysteine to cysteine. Abbreviations: CBS, cystathionine-β-synthase; CGL, cystathionine-γ-lyase, EAAT3, excitatory amino acid transporter-3; GCLM, γ-glutamylcysteine ligase modulatory subunit; GR, glutathione reductase; GSH, glutathione; GSS, glutathione synthetase; GSSG, glutathione disulfide; G6PD, glucose-6-phosphate dehydrogenase; Keap1, kelch-like ECH-associated protein 1; Maf, musculoaponeurotic fibrosarcoma proteins; MAT, methionine adenosyltransferase; MS, methionine synthase; MT, methyltransferase; Nrf2, nuclear factor (erythroid-derived 2)-like 2; SAHH, S-adenosylhomocysteine hydrolase.

Nuclear factor (erythroid-derived 2)-like 2 is a cap-n-collar basic region leucine zipper transcription factor. Under normal conditions, Nrf2 is sequestered through its Neh2 domain by a ubiquitin ligase substrate adaptor called Kelch-like ECH-associated protein 1 (Keap1) (13) (Figure 1). Upon interacting with electrophiles, such as reactive oxygen species (ROS), Keap1 releases Nrf2, which relocates to the nucleus, where it heterodimerizes with musculoaponeurotic fibrosarcoma proteins and binds to antioxidant response elements (AREs) with the core consensus sequence 5′-TGACnnnGC-3′ (14). In this way, Nrf2 regulates ≥1% of genes (15). ARE-regulated genes are typically involved in the production of antioxidants (e.g., glutathione) and detoxification enzymes (e.g., glutathione peroxidase 2).

Nuclear factor (erythroid-derived 2)-like 2 acts as a tumor suppressor or an oncoprotein (16), depending upon the nature of the cell expressing it. In normal physiologic conditions, Nrf2 activation is brief and its transcriptional effects can restore redox homeostasis (13). The tight regulation of Nrf2 reduces the risk of cellular transformation from a benign state to a malignant state, reflecting its status as a tumor suppressor in typical cells. This tumor-suppressing effect is attributed to the ability of Nrf2 to decrease ROS levels and to restrict DNA damage by promoting the expression of antioxidant and detoxification systems (13). Although Nrf2 generally suppresses cellular transformation, it may actually enhance this process in some situations. This appears to be the case in BEAS-2B lung bronchial epithelial cells chronically exposed to the metal cadmium (17). In these cells, Nrf2 binds to AREs on the antiapoptotic genes Bcl-2 and Bcl-xL and to an ARE at p62, which codes a ubiquitin-binding protein (17). Phosphorylated p62 maintains constitutive Nrf2 activity in transformed cancer cells, such as transformed BEAS-2B cells, by binding to Keap1 and blocking its suppressive interaction with Nrf2 (17, 18). Consequently, the transcription of ARE-containing genes is increased and loses responsiveness to intracellular redox status in malignant cells. In this setting, Nrf2 promotes tumor cell proliferation by acting as an oncoprotein and increasing tumor resistance to chemotherapy and radiation (19).

The expression of Nrf2-regulated genes is essential for the survival of many cancer types because high levels of ROS are produced during cell growth, implying that cancer cells are burdened with higher levels of ROS than non-neoplastic cells (20). Higher ROS levels can impair enzyme function, and several enzymes involved in epigenetic regulation are especially sensitive to ROS, including enzymes discussed in this article: MS, MCM, histone deacetylases (HDACs) 2 and 4 (HDAC2/4) (21, 22), and propionyl-CoA carboxylase (23).

Mitochondrial respiration is a major source of ROS production and increased antioxidant capacity secondary to Nrf2 activation increases mitochondrial aerobic activity, while Nrf2 interaction with other transcriptional factors increases mitochondrial biogenesis. Indeed, Nrf2 and Keap1 are tethered to the outer mitochondrial membrane phosphatase phosphoglycerate mutase family member PGAM5, representing a third site of distribution (beyond cytoplasmic and nuclear) (24). PGAM5 degradation is in turn regulated by a Keap1-dependent ubiquitin ligase complex (25), indicating that Nrf2/Keap1 and mitochondrial activities are coordinated.

Nuclear factor (erythroid-derived 2)-like 2 is overexpressed in glioblastoma (26) and elevated expression of genes under the transcriptional influence of Nrf2 occurs in 13.7 and 32.7% of anaplastic gliomas and glioblastomas, respectively (27). There is a strong connection between ROS (and other radicals) and the epigenetic mechanisms that regulate gene expression. Because Nrf2 is a central regulator of the intracellular balance between antioxidants and free radicals, its overexpression may account for much of the epigenetic divergence of glioblastoma from its precursor cells.

## Nrf2, Antioxidants, and Cobalamins

Cobalamin is a corrin ring-containing structure with a central cobalt atom. Different cobalamin species have a variable upper axial ligand bound to the cobalt, enabling cobalamin to act as a cofactor for two enzymes in humans. MeCbl is utilized by MS, while AdoCbl is necessary for the activity of MCM. Cobalt contributes to the propensity of cobalamin to be a highly potent reducing agent, and thus an easily oxidized molecule.

For cobalamin to be converted into its active forms, the cobalt atom must be in its (II) transition state (28). This is achieved enzymatically after the cellular uptake of cobalamin in a complex with transcobalamin, a cobalamin-carrier protein (29). The complex is initially imported into cells *via* the transcobalamin receptor (30). As cobalamin accumulates in tumors (31), both transcobalamin and transcobalamin receptor have been explored as potential tumor biomarkers and their expression is elevated in numerous canine and feline cancers (32). Cobalamin exits lysosomes and interacts with a cytoplasmic enzyme, methylmalonic aciduria, and homocystinuria type C protein (MMACHC), that removes the upper axial ligand and reduces the cobalt atom to its (II) oxidized state (29). To accomplish this, MMACHC uses NADPH to remove cyano groups (from cyanocobalamin) and reduced glutathione (GSH) to remove methyl and adenosyl groups (33).

**Figure 2:**
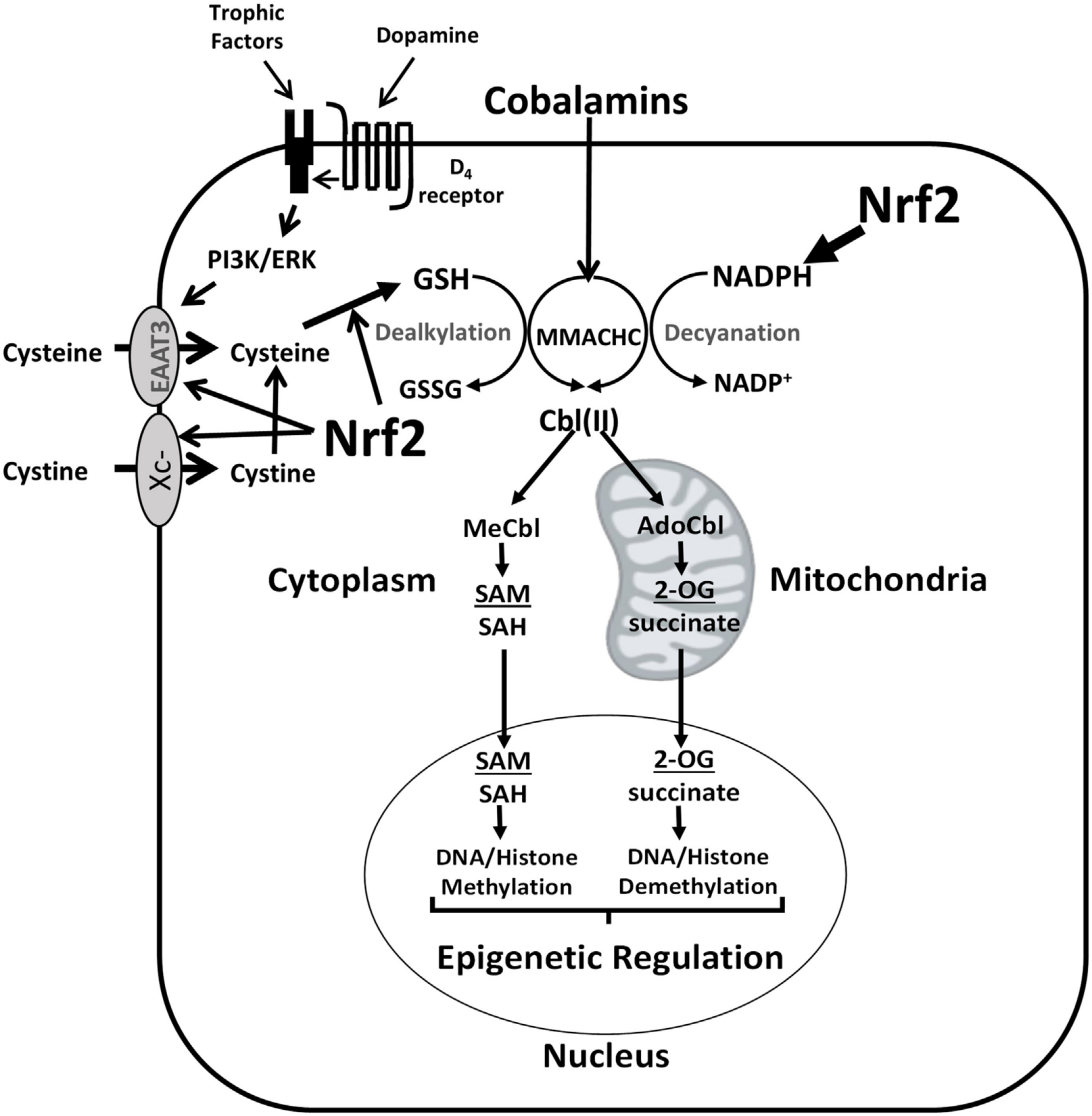
**Nrf2 regulation of cobalamin metabolism**. Nrf2 upregulates Xc^−^ and EAAT3 transcription, increasing cysteine availability for GSH synthesis. Nrf2 also upregulates the formation of GSH and NADPH, promoting MMACHC-dependent conversion of cobalamin to its active forms (MeCbl and AdoCbl). Mutations in PI3K- and ERK-linked trophic receptors (e.g., epidermal growth factor receptor and platelet-derived growth factor receptor beta), can increase their activities, augmenting cystine/cysteine uptake and increasing the levels of active cobalamin species, with epigenetic consequences. Transactivation of PDGFR by the D_4_ receptor also promotes PI3K/ERK signaling and cystine/cysteine uptake. Abbreviations: 2-OG, 2-oxoglutarate; AdoCbl, adenosylcobalamin; Cbl, cobalamin; EAAT3, excitatory amino acid transporter-3; GSH, glutathione; GSSG, glutathione disulfide; MeCbl, methylcobalamin; MMACHC, methylmalonic aciduria and homocystinuria type C protein; Nrf2, nuclear factor (erythroid-derived 2)-like 2; PI3K, phosphoinositide 3-kinase; SAH, S-adenosylhomocysteine; SAM, S-adenosylmethionine.

The potential for Nrf2 to regulate cobalamin status is related to its ability to transcriptionally activate enzymes involved in regulating the intracellular concentrations of the metabolic reducing agents (i.e., GSH and NADPH), which are necessary to process and protect cobalamins. Indeed, Nrf2 may be a key factor that increases the cobalamin content of glioblastoma cells in response to activation of the phosphoinositide 3-kinase (PI3K) or extracellular signal-regulated kinase (ERK) pathway induced by growth factor receptor signaling, irrespective of whether these pathways are stimulated by their respective ligands or transactivated by D_4_ receptors (34). Of note, some mutations may result in hyperactivity of these transduction pathways (Figure 2). In SH-SY5Y neuroblastoma cells, stimulation of the PI3K pathway by the growth factor neuregulin-1 was associated with increased intracellular concentrations of cobalamin owing to activation of the cysteine transporter excitatory amino acid transporter-3 (EAAT3) (35). EAAT3 translocation to the plasma membrane in response to PI3K activation is associated with an increase in GSH, and Nrf2 is a known transcriptional activator of EAAT3 (36). PI3K and ERK are known to promote the activity of Nrf2 in glioblastoma (37), which may increase cobalamin levels by upregulating NADPH and GSH.

Excitatory amino acid transporter-3 levels are increased by neuregulin-1 in C6 glioma cells (38), a cell line that serves as a useful model for the study of glioblastoma. The cystine/glutamate antiporter, also known as system Xc^−^, provides an important source of cysteine for GSH synthesis, as cystine is reduced to two cysteine molecules in the cytoplasm. Nrf2 induces transcription of xCT (SLC7A11), a component of Xc^−^, along with other GSH synthesis and redox-related genes (Table 1).

**Table 1:** **Genes and corresponding proteins regulated by Nrf2**.

Gene	Protein/enzyme name	Role	Reference
*SLC1A1*	Excitatory amino acid transporter 3	Cysteine transporter, usually associated with neurons	(36)
*GCLC*	γ-glutamylcysteine ligase (catalytic subunit)	Subunits of the rate-limiting enzyme for GSH production	(39)
*GCLM*	γ-glutamylcysteine ligase (modifier subunit)		
*TXNRD1*	Thioredoxin reductase	Selenoprotein in thioredoxin antioxidant pathway	(40, 41)
*G6PD*	Glucose-6-phosphate dehydrogenase	Pentose phosphate pathway enzyme that maintains reduced NADPH	(42)
*SLC7A11*	xCT subunit of amino acid transporter system Xc^−^	Cystine transporter in astroglial cells	(43, 44)
*HSP70*	Heat shock protein 70	Cellular stress response protein	(45)
*HSP40*	Heat shock protein 40		
*MTHFD2*	Methylenetetrahydrofolate dehydrogenase 2	Purine synthesis	(46)
*IDH1*	Isocitrate dehydrogenase 1	Converts isocitrate to 2-oxoglutarate, regenerating NADPH	(47)

Epidermal growth factor receptor activation in C6 glioma cells upregulates xCT in association with increased GSH levels (48). In mice, EGFR-overexpressing gliomas are associated with greater invasiveness, and the xCT inhibitor sulfasalazine suppressed tumor growth and invasiveness. Taken together, these findings suggest that increased EGFR signaling may increase xCT and GSH synthesis *via* increased Nrf2 expression, contributing to the aggressive properties of glioblastoma.

## Nrf2, SAM, and Glioblastoma

Many biological processes are regulated by methylation reactions, including DNA and histone methylation. These reactions are governed by the availability of the universal methyl donor SAM, and methyltransferases are inhibited by the demethylated end-product S-adenosylhomocysteine (SAH). The SAM to SAH ratio is affected by the kinetics of the cobalamin- and folate-dependent enzyme MS, which converts homocysteine to methionine.

While normal cells can adapt to a lack of methionine, many cancer cell lines require methionine, as demonstrated by their inability to survive just on methionine provided by MS activity (i.e., methionine derived from homocysteine) (49). While the cause of this condition varies, it may reflect a limitation in cobalamin or folate availability. By contrast, methionine-independent cancer cells can maintain sufficient levels of MS activity and methionine synthesis to sustain their rapid growth rates.

**Figure 3:**
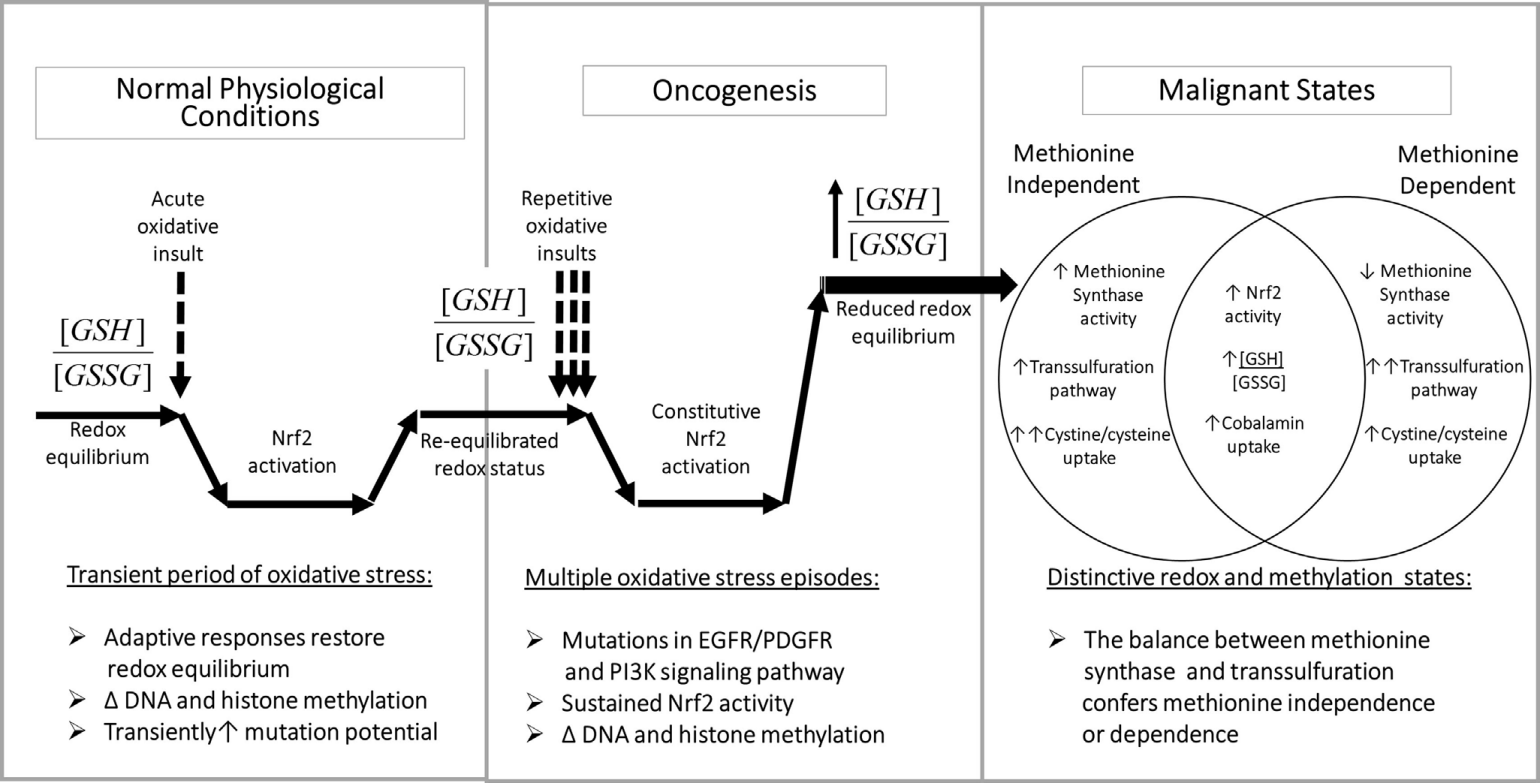
**Redox-dependent epigenetic regulation and oncogenesis**. Left panel: transient Nrf2 activation is an important component of the adaptive metabolic responses to oxidative stressors, accompanied by epigenetic changes, which promote homeostatic restoration of redox equilibrium. Middle panel: multiple oxidative challenges increase the potential for DNA mutations, including mutations of growth factor signaling pathway components, which can lead to sustained Nrf2 activation, resulting in abnormal redox and epigenetic status. Right panel: methionine-independent and methionine-dependent states can alternatively emerge from oncogenesis, depending upon the level of MS activity, which is influenced by the ratio of cystine/cysteine uptake vs. transsulfuration. Abbreviations: GSH, glutathione; GSSG, glutathione disulfide; MS, methionine synthase; Nrf2, nuclear factor (erythroid-derived 2)-like 2.

By augmenting GSH synthesis, Nrf2 activity can influence methionine dependence and the cell’s methylation state in multiple ways. As illustrated in Figure 3, left panel, the normal adaptive response to oxidative stress involves a transient increase in Nrf2 activity together with a decrease in the ratio of reduced GSH to oxidized glutathione disulfide (GSSG) (i.e., [GSH]^2^/[GSSG]). Increased transcription of ARE-regulated genes serves to augment antioxidant capacity, restoring redox status and allowing Nrf2 activity to return to its basal level. However, methylation activity decreases during periods of elevated oxidative stress, affecting DNA and histone methylation, with the potential for persistent epigenetic effects.

The potential for DNA mutations increases during transient oxidative stress (due to unquenched oxidizing species) and/or increased prevalence of euchromatin (non-compacted DNA). Cancer-inducing mutations in pathways controlling Nrf2 expression (e.g., PI3K) during repetitive or unresolved periods of oxidative stress can lead to constitutive Nrf2 activation (Figure 3, middle panel). Unlike in normal physiologic conditions, mutation-induced Nrf2 overexpression is not transient and can lead to an abnormally high antioxidant capacity, which promotes cell division and produces an abnormal pattern of DNA and histone methylation.

As illustrated in Figure 3 (right panel), sustained Nrf2 hyperactivity induced by mutations can promote methionine-independent or methionine-dependent phenotypes, depending on the level of MS activity. Under conditions where the increase in cystine/cysteine uptake is dominant, MS-mediated methylation of homocysteine is maintained, resulting in methionine independence. However, when the increase in transsulfuration of homocysteine toward GSH production is more dominant, MS activity is insufficient, resulting in methionine dependence. Thus, the balance between cystine/cysteine uptake and homocysteine transsulfuration is a major determinant of methionine dependence.

Increased cystine/cysteine uptake capacity secondary to Nrf2 activation may have implications on the tumor microenvironment. Increased expression of Xc^−^ and EAAT3 enhances tumor uptake of antioxidant resources at the expense of other cells in the microenvironment. Glioblastoma cells may competitively impinge on EAAT3-dependent cysteine uptake by neuronal cells, with potential functional implications for neurons.

Nuclear factor (erythroid-derived 2)-like 2 can also affect MS activity by influencing cobalamin synthesis and cellular redox status. As described above, Nrf2 can promote cobalamin assimilation through its ability to increase GSH and NADPH level, and the resultant increase in cobalamin availability decreases methionine dependency by sustaining MS activity (50). In addition, a Nrf2-induced decrease in oxidative stress status decreases the frequency of cobalamin inactivation and promotes MS activity. When the cobalt atom of the cobalamin cofactor of MS is in its (I) oxidation state, it removes and transiently holds a methyl group from 5-methyltetrahydrofolate. The methyl group is subsequently transferred to homocysteine, forming methionine. However, cobalamin (I) is readily oxidized, depending upon the prevailing redox environment, temporarily halting enzyme activity, until it is reactivated by NADPH-dependent MS reductase. Thus, hyperactivity of Nrf2 decreases the rate of cobalamin oxidation by lowering oxidative stress and ROS levels, and facilitates MS reactivation by promoting higher levels of NADPH.

The availability of methionine is proportionally reflected by intracellular SAM concentrations (51). Therefore, the involvement of Nrf2 in maintaining a methionine-independent state may dictate how glioblastoma cells allocate SAM toward different reactions. SAM-dependent methylation reactions in the nucleus control gene expression through multiple mechanisms. Thus, the availability of SAM, as modulated by Nrf2, may affect the survival and proliferation of glioblastoma cells by influencing genome-wide gene transcription.

In addition to its role in DNA and histone methylation, SAM is used for the synthesis of polyamines, which are involved in cell growth, transcription, translation, and numerous other processes (52). Indicative of the importance of polyamines in cancer, a polyamine analog was reported to induce apoptosis in glioblastoma cells by exhausting the cellular polyamine content (53). Polyamines are recycled back into the one-carbon cycle as methylthiooxobutyrate to re-form methionine and subsequently SAM. SAM may also influence many processes involved in the progression of glioblastoma, including cell signaling, cell cycle progression, protein translation, mRNA splicing, and possibly the health of telomeres (54).

Finally, SAM may influence glioblastoma by acting as a positive allosteric modulator of proteins involved in maintaining redox homeostasis, including cystathionine-β-synthase (CBS) and Nrf2. CBS is one of two Nrf2-regulated enzymes in the transsulfuration pathway. This pathway directs homocysteine away from the one-carbon cycle toward cysteine synthesis, accompanied by α-ketobutyrate formation (Figure 1). Notably, Nrf2 is post-translationally methylated at an arginine residue (R437) in its Neh1 domain by the arginine methyltransferase PRMT1 (55). R437 methylation of Nrf2 increases its DNA-binding ability and increases recruitment of transcriptional coactivator p300/CBP (CREB-binding protein) to an ARE in the heme oxygenase-1 promoter region. However, expression of both Nrf2 and its suppressor keap1 are also subject to epigenetic regulation by promoter methylation in several cancers types (56). Thus, the relationships between Nrf2, SAM, and glioblastoma are highly complex.

## Nrf2 and Oncometabolites in Glioblastoma

Oncometabolites are metabolic intermediates that promote tumor growth and other malignant processes. These oncogenic metabolites are either produced excessively or are inefficiently metabolized in cancer and consequentially accumulate in the cytosol (57). Well-known examples of oncometabolites include succinate and fumarate. Mutations in succinate dehydrogenase (SDH) and fumarate hydratase prevent the passage of their respective substrates, succinate and fumarate, through the Krebs cycle, resulting in their accumulation. Mutations in these proteins have been linked to malignant pheochromocytoma, paraganglioma, and other forms of cancer (58–60). When mutated in glioblastoma, IDH1/2 yield another oncometabolite, 2-hydroxyglutarate (2-HG), from 2-oxoglutarate (2-OG) (also known as α-ketoglutarate) (61). While mutations in IDH1/2 are clinically relevant (62), only IDH1 is transcriptionally regulated by Nrf2.

Because Nrf2 enhances cobalamin processing, and thus AdoCbl levels, it can connect the transsulfuration pathway to the Krebs cycle. AdoCbl promotes the formation of succinate. As a cofactor for the mitochondrial enzyme MCM, AdoCbl is necessary for the catabolism of certain fats and amino acids in a pathway involved in the anaplerotic incorporation of succinate into the Krebs cycle as succinyl-CoA, which is then converted to succinate.

The amino acid methionine is catabolized through this anaplerotic pathway. The passage of methionine through the one-carbon cycle produces homocysteine, and any homocysteine that is not recycled back to methionine can enter the transsulfuration pathway and be converted to cystathionine. Cystathionine is then converted to cysteine and α-ketobutyrate. While cysteine is directed toward GSH production, α-ketobutyrate is converted to propionyl-CoA, which is subsequently converted to d-methylmalonyl-CoA by the biotin-dependent enzyme propionyl-CoA carboxylase. d-methylmalonyl-CoA is converted to l-methylmalonyl-CoA by epimerase, and MCM (with its AdoCbl cofactor) converts l-methylmalonyl-CoA to succinyl-CoA.

The product of this pathway, succinyl-CoA, enters the Krebs cycle and is converted to succinate by succinyl-CoA synthetase. Succinate is subsequently converted to fumarate by SDH, or complex II of the mitochondrial electron transport chain. Fumarate and succinate can act as retrograde signaling messengers, relaying information from the mitochondria through their influence over epigenetics and cell signaling. Both fumarate (63) and succinate (64) inhibit prolyl hydroxylases from ubiquitinating hypoxia-inducible factor-1α (HIF-1α) and allow its buildup in normoxic conditions. However, only fumarate binds to cysteine residues on keap1, increasing the amount of Nrf2 available to promote gene transcription (65). SDH subunit B was downregulated and HIF-1α was expressed in glioblastoma stem cells in normoxic conditions (66). However, certain glioblastoma-associated mutations promote oxidative phosphorylation rather than glycolysis (67). Therefore, the amount of succinate and fumarate in glioblastoma cells may vary according to how closely they resemble a stem cell phenotype and the presence of specific mutations in energy producing pathways, in addition to AdoCbl levels; factors that may be influenced by Nrf2.

Nuclear factor (erythroid-derived 2)-like 2 can increase the level of 2-HG, another retrograde signaling molecule in glioblastoma. 2-HG is produced by mutant forms of IDH1/2. Normally, IDH1/2 promote the conversion of isocitrate to 2-OG, while reducing NADP^+^ to NADPH. However, mutated forms of IDH1/2 convert 2-OG to 2-HG. Furthermore, these mutant forms oxidize NADPH to NADP^+^. Ordinarily, the expression of IDH2 (the mitochondrial isoform) is not directly induced by Nrf2. However, the activity of mutated IDH2 may be indirectly promoted by Nrf2 through the increased concentration of NADPH. Around 80% of low-grade gliomas and 5–10% of glioblastomas have IDH1/2 mutations (3) and display increased methylation in the gene promoters compared with other glioma subtypes.

## Epigenetic Effects of 2-HG, Succinate, and Fumarate

Epigenetic control of gene expression is mediated by numerous regulatory factors and represents an overlaying program that governs the manner in which the genetic code is read. Specifically, epigenetic alterations determine the probability of gene expression, the timing of expression, and which isoforms of a specific gene are produced. One of the most important epigenetic events is the chemical modification of amino acid residues in histone tails by ubiquitin, acetyl, phosphoryl, and methyl groups. Histone tail methylation marks are laid down by histone methyltransferases. These marks include H3K4me (which leads to chromatin opening and is positively associated with gene expression), as well as H3K9me and H3K27me (which lead to chromatin condensation and are associated with inactivation of gene expression).

Histone lysine demethylases (KDMs) remove methyl groups from lysine residues on histone tails. There are two classes of KDMs: 2-OG-dependent dioxygenases (2-OGDOs), which possess Jumonji C domains, and flavin-containing amino oxidases. 2-OGDOs can oxidize numerous substrates, which are not limited to methylated lysine and cytosine residues (68). 2-OGDO enzymes convert 2-OG to succinate and CO_2_. 2-OGDO activity is downregulated in a negative feedback loop by succinate, fumarate, and by 2-HG (68).

KDM2-7 are inhibited by the same, whereas KDM1a belongs to the flavin-containing amino oxidase subclass and is not inhibited by these oncometabolites. KDM1a regulates the transcription of embryonic transcription factors by altering Myc expression (69). The role of KDM1a in removing histone tail methylation marks may become more prominent following oncometabolite-mediated inhibition of KDM2-7. Although KDM2-7 are sensitive to succinate, fumarate, and 2-HG, KDM1a activity is regulated by flavin adenine dinucleotide. The reaction mediated by SDH results in the conversion of succinate to fumarate, in which flavin adenine dinucleotide is used as a cofactor to accept two hydrogen atoms and two electrons. Therefore, SDH may act as a junction that regulates both KDM1a and KDM2-7. However, more studies are needed to elucidate the dynamics between SDH, KDM1a, KDM2-7, and histone tail marks in glioblastoma.

Other members of the 2-OGDO family include the TET enzymes. TET enzymes catalyze the first step of cytosine nucleotide demethylation by converting 5-methylcytosine to 5-hydroxymethylcytosine (5hmeC) (70). This step is essential for demethylating CpG sites to allow gene reactivation because methyl-binding proteins cannot block access to transcriptional machinery following demethylation.

Like KDM2-7, TET1-3 are inhibited by succinate, fumarate, and 2-HG. The levels of 5hmeC are low in many cancers, including glioblastoma. A deficit in 5hmeC may indicate a significant reduction in TET enzyme activity that might arise from gene deletions or mutations (71). However, genetic sequencing does not support the hypothesis that mutations are a major causal factor of 5hmC depletion in colorectal, breast, and prostate cancer (72). The expression of mutant IDH1 in the subventricular zone of mice was associated with decreased 5hmeC genomic content and increased 5-methylcytosine levels, mimicking gliomagenesis (73). Therefore, it is plausible that oncometabolite-mediated inhibition of TET activity contributes to the reduced 5hmeC expression observed in these cancers and in glioblastoma.

## Redox and Transcriptionally Induced Epigenetic Effects of Nrf2

Nuclear factor (erythroid-derived 2)-like 2 can exert epigenetic effects *via* multiple mechanisms. As described in the Section “The Role of Nrf2 in Cancer and Glioblastoma,” Nrf2 influences the transcription of ARE-containing genes to increase the levels of antioxidants and 2-HG (in IDH1 mutants). This increase in antioxidant levels protects cobalamin from oxidation, sustaining the one-carbon cycle. Antioxidants also protect the enzyme propionyl-CoA carboxylase, which is upstream of MCM in an anaplerotic pathway that leads to the formation of succinate. The metabolic information conveyed by the intracellular concentrations of SAM and the oncometabolites succinate and 2-HG is integrated at the nucleus and determines global DNA and histone methylation and demethylation.

While Nrf2 indirectly regulates epigenetics through chemical messengers, such as succinate and 2-HG, it can also regulate epigenetics in a more direct manner. Many epigenome-modifying proteins are sensitive to the intracellular redox balance, the prime examples being HDACs 2 and 4 (21, 74). HDACs remove acetyl groups from histone tail residues and non-histone proteins, thereby decreasing gene transcription. HDAC4 expression is downregulated in glioblastoma compared with other gliomas and normal brain tissue, although there is considerable variation in its expression levels between resected tumors from glioblastoma patients and other gliomas (75). The protective effect of Nrf2 on HDAC4 was reported to precede HDAC4-mediated downregulation of the microRNAs miR-206 and miR-1 in lung and prostate cancer cell lines (22). Downregulation of these microRNAs by HDAC4, downstream of Nrf2, increased the expression of Krebs cycle and pentose phosphate pathway components. These findings support the hypothesis that the epigenetic effects of Nrf2 have redox and metabolic consequences.

## D_4_ Receptors, Methylation, and Glioblastoma

Dolma et al. (12) recently reported that selective D_4_ receptor antagonists restrict the growth of glioblastoma stem cells *in vitro* and *in vivo*. Clonogenic activity of freshly isolated tumor cells was reduced 19- to 83-fold by these antagonists. Analysis of gene expression in cells exposed to a D_4_ receptor antagonist revealed significant changes in lipid/cholesterol biosynthetic, autophagic vacuole, and lysosomal pathways, leading massive accumulation of autophagic vacuoles and cholesterol owing to decreased autophagic flux and disrupted lysosomal function. These glioblastoma stem cell-specific effects were accompanied by decreased phosphorylation of multiple proteins, including platelet-derived growth factor receptor beta (PDGFRβ), which was previously shown to be transactivated by D_4_ receptor stimulation (34). Indeed, transactivation of PDGFRβ and its downstream signaling target ERK1/2 by the D_4_ receptor may contribute to increased Nrf2 activity, as described above, while D_4_ receptor antagonists have opposite effects. Since PDGFRβ levels are increased in proneural glioblastoma, more robust D_4_ receptor co-activation may be particularly important in this subtype. The inhibitory effects of D_4_ receptor antagonists were synergistic with those of temozolomide, and poorer survival rates were observed for tumors with greater D_4_ receptor expression.

Although the study by Dolma et al. (12) showed that the D_4_ receptor is an important new antitumor target for glioblastoma, the detailed molecular mechanism underlying its unique autophagy-based effectiveness remain unclear. Like other GPCRs, the D_4_ receptor exerts several G protein-mediated signaling activities, including inhibition of cyclic AMP formation. However, only the D_4_ receptor is involved in dopamine-dependent methylation of membrane phospholipids (76). This exclusive activity arises from a methionine residue (MET313) in transmembrane helix #6 of the D_4_ receptor, not present in other GPCRs, a locus that undergoes a large change in position in response to agonist occupation of the binding site (77). This dopamine-induced conformational change facilitates ATP-dependent S-adenosylation of the MET313, followed by transfer of its methyl group to adjacent phospholipids, especially phosphatidylethanolamine (78). After methyl transfer and removal of the adenosyl group, a replacement methyl group is provided to the resulting homocysteine form of the receptor by MS, in a cobalamin/methylfolate-dependent reaction, completing a cycle of dopamine-stimulated phospholipid methylation (PLM).

**Figure 4:**
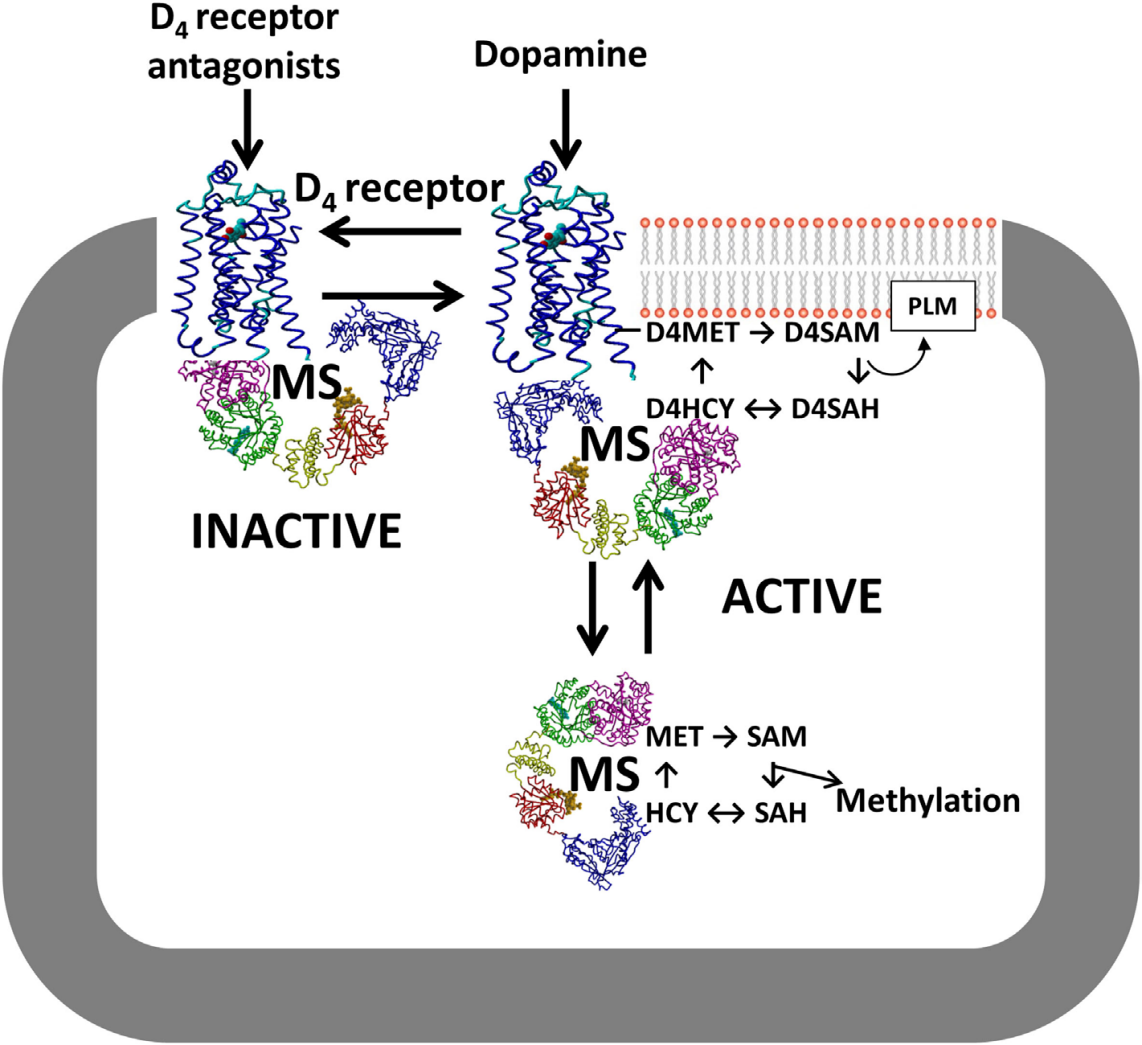
**D_4_ receptor interaction with MS**. Conformation-dependent exposure of the methionine residue in the D_4_ receptor allows it to catalyze phospholipid methylation (PLM), which is dependent upon folate-derived methyl groups supplied by MS. D_4_ receptor antagonists stabilize the inactive conformation and sequester MS, while D_4_ receptor agonists such as dopamine promote PLM and increase MS-dependent conversion of HCY to MET. Abbreviations: D_4_R, D_4_ receptor; HCY, homocysteine; MET, methionine; MS, methionine synthase; SAH, S-adenosylhomocysteine; SAM, S-adenosylmethionine.

Notably, the cycle of D_4_ receptor-mediated PLM utilizes the same enzymes that support SAM-dependent methylation reactions (76), placing the D_4_ receptor in the position of regulating all methylation reactions in a dopamine-dependent manner, as illustrated in Figure 4. Indeed, D_4_ receptor-mediated stimulation of MS activity and increased global DNA methylation have been reported in human neuroblastoma cells (79), and these effects were blocked by inhibitors of the PI3K and ERK signaling pathways. It remains to be determined whether D_4_ receptors exert a similar influence in glioblastoma.

Based on the unique capability of the D_4_ receptor to modulate methylation status, we suggest that the anti-glioblastoma tumor effects of D_4_ receptor antagonists described by Dolma et al. (12) may reflect inhibition of methylation reactions such as dopamine-stimulated PLM, which can influence the activity of receptors and transporters at the plasma membrane (80), and/or SAM-dependent methylation effects, which might include effects on autophagy. For example, increased membrane fluidity and/or membrane asymmetry promoted by D_4_ receptor-mediated PLM may contribute to PDGFRβ transactivation. PLM-related phospholipid adducts were also reported to play crucial regulatory roles in promoting mitophagy, a specialized form of autophagy (81). At high levels, methionine and SAM act as indicators of amino acid sufficiency and suppress autophagy (82). This hypothesis is supported by the finding that autophagy was attenuated by short hairpin RNA-mediated knockdown of MS reductase in SKOV3 and cisplatin-resistant SKOV3/DDP cells (83). Studies of dopamine-stimulated PLM may help our understanding of how the D_4_ receptor regulates autophagy in glioblastoma stem cells.

## Potential Treatment Targets

Despite the development of temozolomide and bevacizumab, glioblastoma invariably has a poor prognosis, necessitating the development of more effective therapies. Several classes of drugs are being explored, including many that target the epigenetic landscape. Some epigenetic modifiers, such as BET proteins, are overexpressed in glioblastoma. Because BET proteins are indispensable for glioblastoma growth (10), BET inhibitors are an important area of research. JQ1 is a BET inhibitor that caused G1 cell cycle arrest and apoptosis in several glioblastoma cell lines, which were genetically modified to resist cell death (84). However, JQ1 has a short half-life, limiting its utility in glioblastoma treatment (85). Another BET inhibitor, OTX015, has entered dose optimization trials for glioblastoma therapy (http://ClinicalTrials.gov Identifier NCT02296476).

Histone deacetylases inhibitors have been also tested in glioblastoma (86), although their clinical use is generally limited to certain lymphomas. HDAC inhibitors reactivate the expression of genes, which either promote cell death or improve the efficacy of other drugs. For example, HDAC inhibitors can restore the expression of estrogen receptor α (ERα) in ERα negative breast cancer, potentially re-allowing the administration of tamoxifen to tumors that had become non-responsive to antiestrogen drugs (87). However, a concern of using HDAC inhibitors for treating glioblastoma is that they might re-activate expression of O^6^-alkylguanine DNA alkyltransferase (MGMT), a DNA repair methyltransferase associated with temozolomide resistance (88).

An interesting approach to circumvent the risk of re-activating MGMT in glioblastoma is to administer drugs capable of modulating the redox capacity, such as the flavonoid luteolin. Luteolin was reported to reduce Nrf2 mRNA and protein levels in tumors (89), thus affecting redox and epigenetic regulation. Owing to the antioxidant-boosting and antiapoptotic effects of Nrf2, the benefits of a Nrf2 inhibitor may outweigh any risks posed by altering the epigenetic signature it imparts to glioblastoma cells. Treatment of A549 cells with luteolin enhanced the cytotoxicity of doxorubicin, oxaliplatin, and bleomycin (89). Furthermore, a combination of luteolin and another flavonoid, silibinin, caused autophagy and apoptosis by inhibiting protein kinase Cα (PKCα) and inducible nitric oxide synthase (iNOS), respectively, in U87MG and T98G glioblastoma cells (90). Additionally, luteolin is considered to be neuroprotective and was ironically demonstrated to induce Nrf2 activity in a mouse model of traumatic brain injury (91). Therefore, this phytochemical may selectively target neoplastic tumor cells and protect surrounding brain tissue.

Because cystine transporter activity is upregulated in glioblastoma (48), selective transporter inhibitors might be useful treatment options. Indeed, several studies have reported beneficial effects of sulfasalazine, an inhibitor of systemic Xc^−^-mediated cystine uptake (92, 93). However, in a small clinical trial, sulfasalazine was without benefit in recurrent glioblastoma (94). In addition, sulfasalazine did not improve the outcomes of combined temozolomide and radiotherapy in patients with newly diagnosed glioblastoma (95). This therapeutic failure may be due to Nrf2-mediated upregulation of Xct induced by temozolomide (96), offsetting the inhibitory effects of sulfasalazine (92). Erastin, another Xct inhibitor, was reported to be superior to sulfasalazine for induction of cell death when administered in combination with temozolomide to C6 glioma cells, owing to its ability to decrease the expression of the Nrf2-induced transsulfuration enzyme cystathionine-γ-lyase (92), thereby depleting GSH formation *via* two mechanisms.

Inhibition of GSH synthesis can be compensated for by Nrf2-induced upregulation of the NADPH-dependent thioredoxin pathway (20). However, combined treatment with inhibitors of GSH synthesis (e.g., buthionine sulfoximine) and thioredoxin reductase (e.g., auranofin) decreased tumor survival in A172 glioblastoma cells (20). Although a combination of GSH and thioredoxin pathway inhibitors was efficacious *in vitro*, it may be unsuitable for patients considering the potential safety and tolerability of this combination. However, it may be possible to limit its toxicity by utilizing targeted drug delivery techniques.

## Conclusion

**Figure 5:**
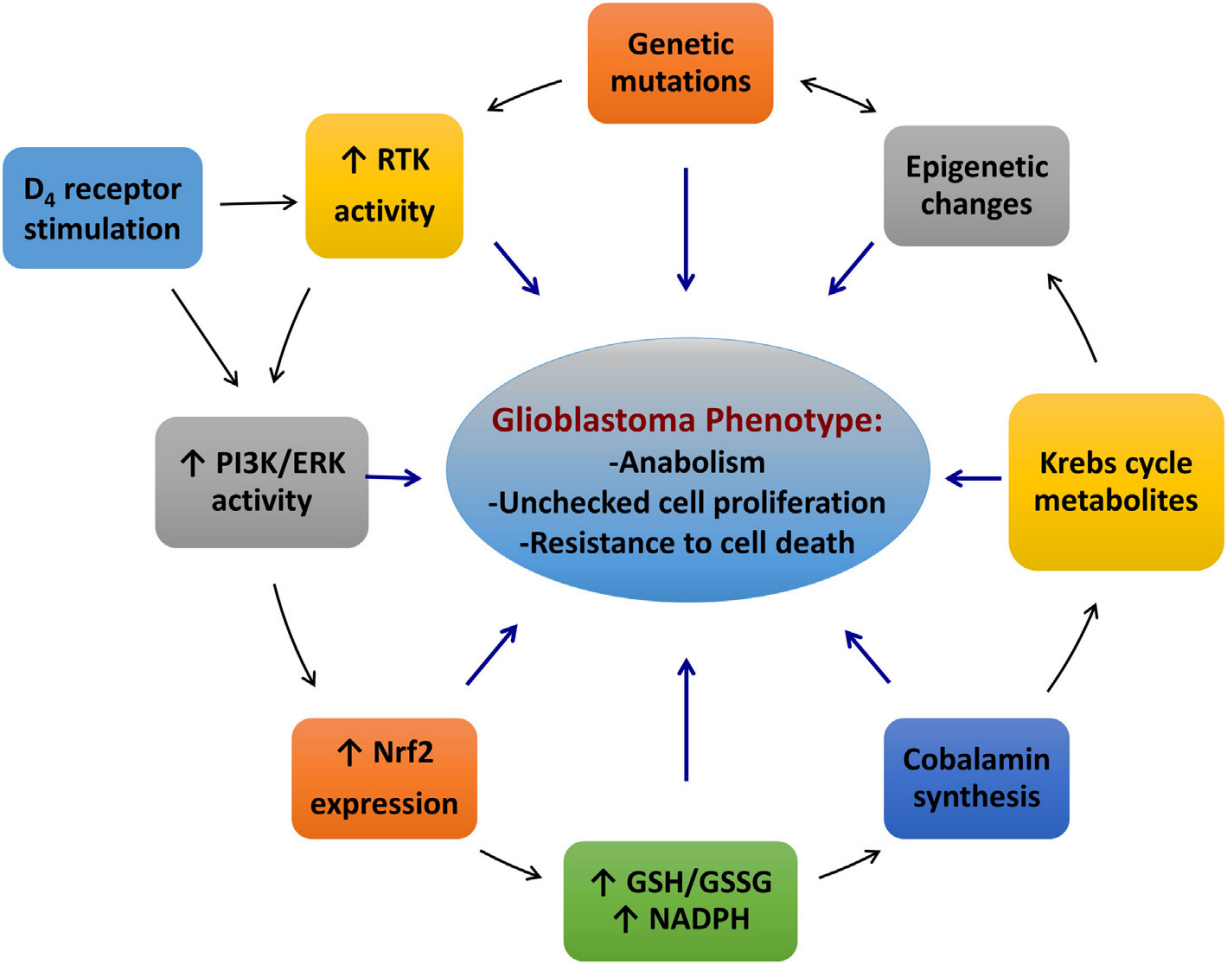
**An interactive network of the redox and methylation factors contributing to the glioblastoma phenotype**. Abbreviations: ERK, extracellular signal-regulated kinase; GSH, glutathione; GSSG, glutathione disulfide; Nrf2, nuclear factor (erythroid-derived 2)-like 2; PI3K, phosphoinositide 3-kinase.

We have discussed the roles of redox and epigenetic regulation in glioblastoma. The findings described here suggest that glioblastoma is sustained by an intricate network of Nrf2, receptor tyrosine kinases, cobalamins and cobalamin-dependent enzymes, along with the D_4_ receptor and other factors, such as cystine/cysteine transporters and TET enzymes (Figure 5). Mutations in receptor tyrosine kinases or their downstream targets can result in hyperactivity of the PI3K and ERK pathways, leading to constitutively higher Nrf2 activity. Higher Nrf2 activity induces overexpression of genes involved in antioxidant production, such as cystine/cysteine transporters and promotes 2-HG formation in IDH1 mutants. Nrf2 can also upregulate its own activity by promoting p62 transcription. The excessively reduced redox state of glioblastoma cells enhances the uptake and processing of cobalamins and promotes the activity of redox-sensitive proteins (i.e., HDAC2). In methionine-independent cells, the cobalamin-dependent enzyme, MS, enhances the formation of SAM, which influences methyltransferase activity. The other cobalamin-dependent enzyme in humans, MCM, promotes the formation of oncometabolites, affecting a wide range of proteins, including TET1-3, KDM2-7, prolyl hydroxylases, and Keap1. D_4_ receptors may contribute to this network by regulating MS activity *via* stimulation of the ERK/PI3K pathways, either directly or indirectly by transactivation of PDGFRβ. These interactions result in an amalgamation of epigenetic changes, which contribute to the induction of the cancer phenotype and drive tumor growth. Indeed, the model described for glioblastoma can be applied to all cancers, and we hope that the novel concepts presented here will spur new research in this context.

Although existing treatment options for glioblastoma have not achieved clinically meaningful remission rates, epigenetic- and redox-modifying agents may augment the repertoire of available life-saving drugs. HDAC inhibitors have demonstrated utility in treating certain forms of cancer. However, these drugs may reactivate a DNA repair mechanism in glioblastoma. BET inhibitors may be immune to this unwanted effect of HDAC inhibitors. Luteolin and related drugs may sensitize glioblastoma cells to the effects of radiotherapy or chemotherapy and mitigate damage to surrounding neurons and non-cancerous glia. The ability of D_4_ receptor antagonists to induce growth arrest and apoptosis of stem cells is an encouraging development and suggests that a better understanding of the relationships between cancer metabolism, redox status, and epigenetics may lead to significantly higher remission rates. Mutations in redox and methylation pathway genes may complement canonical mutations for existing glioblastoma subtypes and could be incorporated into a panel for pharmacogenomic screening for the optimization of drug treatment. We envision an expansion in the near future of drugs targeting antioxidant production and related biochemical and molecular pathways, offering hope of improved treatment outcomes for glioblastoma.

## Author Contributions

MS, MT, and RD reviewed relevant literature, drafted and revised the manuscript, designed the figures, and read and approved the final manuscript.

## Conflict of Interest Statement

The authors declare that the research was conducted in the absence of any commercial or financial relationships that could be construed as a potential conflict of interest.
